# Myostatin Overexpression and Smad Pathway in Detrusor Derived from Pediatric Patients with End-Stage Lower Urinary Tract Dysfunction

**DOI:** 10.3390/ijms24054462

**Published:** 2023-02-24

**Authors:** Souzan Salemi, Larissa J. Schori, Tim Gerwinn, Maya Horst, Daniel Eberli

**Affiliations:** 1 Laboratory for Urologic Oncology and Stem Cell Therapy, Department of Urology, University Hospital Zürich, 8952 Schlieren, Switzerland; 2 Division of Pediatric Urology, University Children’s Hospital Zürich, 8032 Zürich, Switzerland

**Keywords:** myostatin, bladder-derived smooth muscle, contractile protein

## Abstract

Cell therapies and tissue engineering approaches using smooth muscle cells (SMCs) may provide treatment alternatives for end-stage lower urinary tract dysfunction (ESLUTD). Myostatin, a negative regulator of muscle mass, is a promising target to improve muscle function through tissue engineering. The ultimate goal of our project was to investigate the expression of myostatin and its potential impact in SMCs derived from healthy pediatric bladders and pediatric ESLUTD patients. Human bladder tissue samples were evaluated histologically, and SMCs were isolated and characterized. The proliferation of SMCs was assessed by WST-1 assay. The expression pattern of myostatin, its pathway and the contractile phenotype of the cells were investigated at gene and protein levels by real-time PCR, flow cytometry, immunofluorescence, WES and gel contraction assay. Our results show that myostatin is expressed in human bladder smooth muscle tissue and in isolated SMCs at gene and protein levels. A higher expression of myostatin was detected in ESLUTD-derived compared to control SMCs. Histological assessment of bladder tissue confirmed structural changes and decreased muscle-to-collagen ratios in ESLUTD bladders. A decrease in cell proliferation and in the expression of key contractile genes and proteins, α-SMA, calponin, smoothelin and MyH11, as well as a lower degree of in vitro contractility was observed in ESLUTD-derived compared to control SMCs. A reduction in the myostatin-related proteins Smad 2 and follistatin, and an upregulation in the proteins p-Smad 2 and Smad 7 were observed in ESLUTD SMC samples. This is the first demonstration of myostatin expression in bladder tissue and cells. The increased expression of myostatin and the changes in the Smad pathways were observed in ESLUTD patients. Therefore, myostatin inhibitors could be considered for the enhancement of SMCs for tissue engineering applications and as a therapeutic option for patients with ESLUTD and other smooth muscle disorders.

## 1. Introduction

Tissue loss and organ failure due to injury or disease are considered major healthcare challenges in terms of frequency, severity and costs [1,2]. The urinary bladder wall consists of 60–70% detrusor muscle, which is composed of smooth muscle cells (SMCs) [3]. In pediatric patients, congenital anomalies of the central nervous system or congenital bladder outlet obstruction can lead to end-stage lower urinary tract dysfunction (ESLUTD). Pathological conditions such as congenital disorders and malignancies in children and adults can lead to ESLUTD [4]. The ESLUTD is characterized by fibrosis of the bladder wall with loss of compliance and increased intravesical pressure, which may lead to renal damage. To date, there is no curative drug therapy, and if medical treatment fails, the current treatment is a surgical enlargement of the bladder using intestinal segments, which is associated with severe short- and long-term complications [5,6,7]. In order to improve the therapy of affected patients, our main focus is on the preservation of the functional quality of the bladder wall and, respectively, the improvement of bladder detrusor SMCs. Tissue engineering (TE) using a patient’s own cells can be a promising alternative for clinical applications and cell therapies. Until now, one of the major problems has been the identification of a reliable source of SMCs for TE purposes. Mature SMCs isolated from tissue have shown limited proliferation capacity during the in vitro expansion [8,9]. Therefore, further research on the improvement of SMC quality and proliferation is urgently needed. Myostatin, also known as growth differentiation factor 8 (GDF8), is a negative regulator of muscle growth and differentiation. It was first described in the skeletal muscle [10] but also identified in other tissues such as the adipose tissue [10], cardiomyocytes of the heart [11], the smooth muscle of the penis and its vasculature [12] as well as in endometrial smooth muscle [13]. As a critical regulator of skeletal muscle mass, the lack of myostatin in knockout mice led to approximately twice the normal muscle mass in these mice [10]. A natural mutation in a child led to extraordinary amounts of muscle in the thighs and upper arms [14]. One of the main pathways of myostatin is through activation of Smad signaling, where myostatin leads to phosphorylation of Smad 2/3 and recruitment of Smad 4, leading to an assembly of Smad 4 with p-Smad 2/3 [15]. In both skeletal and smooth muscle there are natural myostatin inhibitors [16] such as the myostatin precursor protein itself [17], follistatin [18,19] and growth and differentiation factor-associated serum protein-1 (GASP-1) [20] that prevent myostatin from binding to its receptor.

Myostatin can be inhibited by antibodies or compounds, which are currently being investigated for their therapeutic potential in clinical trials such as muscle atrophy associated with cancer and muscular dystrophy [21,22,23]. The impact of myostatin extends beyond skeletal muscle proliferation, its alteration contributes to muscle atrophy and cachexia [24], muscle wasting in the chronic obstructive pulmonary [25] and kidney diseases [26]. In addition, elevated myostatin levels were detected in patients with end-stage liver [27] and cardiac diseases [28].

Although the importance of myostatin has been widely studied in skeletal muscle and other tissues, its expression and role were never investigated in bladder-derived smooth muscle. Myostatin inhibition leads to improved muscle cell growth and differentiation and, therefore, can be exploited in a TE strategy [21]. It is known that myostatin-mediated human skeletal muscle atrophy is associated with sarcomeric protein loss, as well as enhanced expression of the ubiquitin E3 ligases atrogin-1 and MURF1 [29]. Since these two proteins are also expressed in smooth muscle tissue, we suppose that myostatin assumes a similar role here as in skeletal muscle [30]. Myostatin inhibition is known to have a huge therapeutic potential to increase muscle size and strength in muscle disorders [31]. However, no information is available on the presence and localization of myostatin in the human bladder detrusor muscle, its potential relationship with the expression of contractile proteins and its regulatory pathways. Therefore, we aimed to investigate the myostatin expression in urinary bladder smooth muscle and its potential impact on bladder SMCs derived from pediatric healthy controls and pediatric ESLUTD patients. 

## 2. Results

### 2.1. Myostatin Expression in Human Bladder 

**Figure 1:**
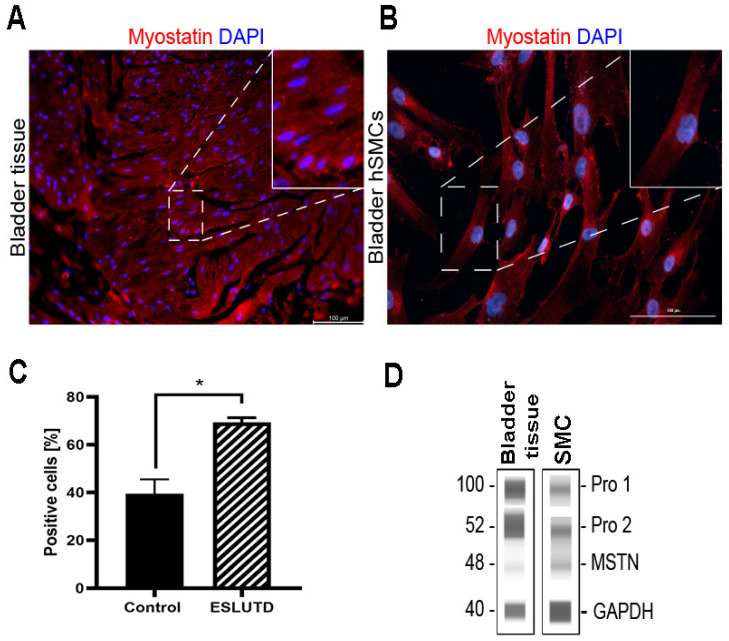
Myostatin expression in human bladder smooth muscle tissue and cells. (**A**) Immunofluorescence staining image of myostatin protein (red) in pediatric bladder muscle tissue. Cell nuclei stained with DAPI (blue). 10× magnification (**B**) Immunofluorescence staining image of myostatin protein (red) in pediatric bladder-derived hSMCs. 20× magnification (**C**) Flow cytometry comparison of myostatin-positive cells in pediatric bladder-derived control and ESLUTD hSMCs. (**D**) Representative immunoblot by WES of active myostatin (48 kDa) and its propeptides (100, 52 kDa) in bladder-derived smooth muscle tissue and cells. GAPDH (40 kDa) as a housekeeping protein. * *p* ≤ 0.05. Scale bar, 100 μm.

The expression of myostatin as a negative regulator of muscle mass has not yet been reported in human bladder smooth muscle tissue and hSMCs. Therefore, our first question was whether myostatin is expressed in human bladder detrusor muscle. Here, we were able to prove the presence of myostatin in human bladder smooth muscle tissue and in hSMCs isolated thereof using different techniques. First, we showed the expression of myostatin protein using immunofluorescence staining in bladder tissue (Figure 1A) and bladder-derived hSMCs (Figure 1B). By flow cytometry, we verified the presence of myostatin in hSMCs (Figure 1C). The ESLUTD-derived hSMCs showed significantly higher expression of myostatin (69.28 ± 2.0 SEM, *p* = 0.03) compared to the healthy control (39.45 ± 6.0). However, the number of positive cells varied greatly between different primary cells. Further, we confirmed myostatin protein expression in both bladder tissue and bladder hSMCs by Simple Western. WES analysis confirmed the presence of mature myostatin dimer at 48 kDa. In addition to the active myostatin dimer, we detected two other bands showing the myostatin propeptides before (~100 kDa) and after (52 kDa) cleavage by proteases in all samples investigated (Figure 1D). 

### 2.2. Comparison of Myostatin Expression in Human Bladder Tissue Derived from Control and ESLUTD Pediatric Patients

**Figure 2:**
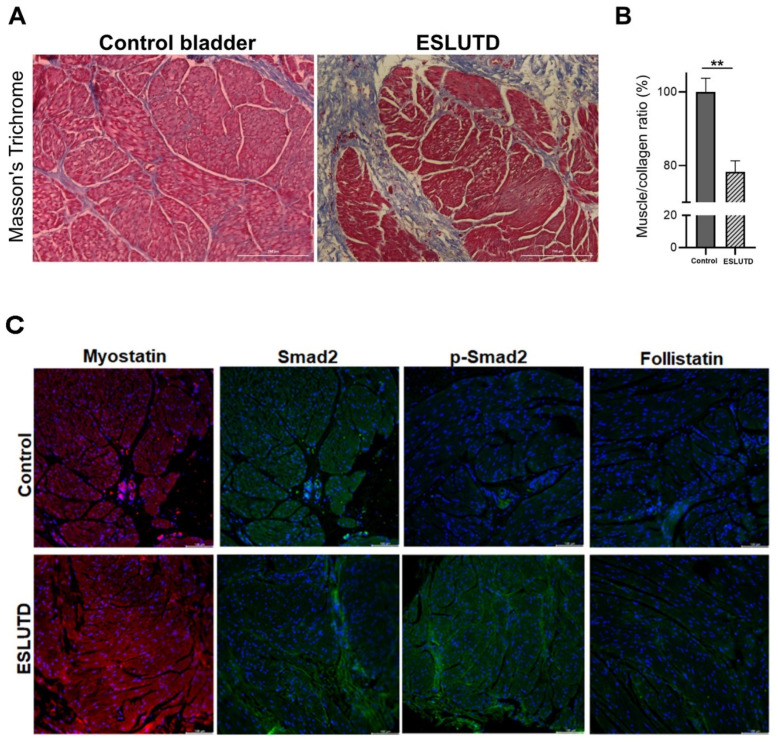
Differences in muscle-to-collagen ratio and Smad pathway proteins in control and ESLUTD bladder. (**A**) Masson’s trichrome staining in control and ESLUTD bladder detrusor muscle with smooth muscle tissue (red) and connective tissue (blue). (**B**) Quantification of muscle-to-collagen ratio in control (n = 4) and ESLUTD (n = 4) smooth muscle tissue, normalized to control taken as 100%. Muscle-to-collagen ratio was significantly lower in ESLUTD compared to control bladder. Statistics with unpaired *t*-test, ** *p* ≤ 0.01. (**C**) Antibody staining for myostatin (red) and its signaling pathway proteins Smad 2 (green), p-Smad 2 (green) and follistatin (green) with cell nuclei in blue. Scale bar, 100 μm.

Histological assessment via Masson’s Trichrome staining of the bladder tissue confirmed the previously described alterations of ESLUTD, such as thinned out, turbulent appearing smooth muscle bundles and declined muscle-to-collagen ratios (Figure 2A,B). In a healthy bladder wall, connective tissue is evident as a thin layer between the smooth muscle bundles. On the contrary, excessive deposits of collagen/connective tissue consistent with fibrosis were observed within the detrusor muscle in ESLUTD (Figure 2A,B). Next, we stained the detrusor muscles with myostatin, Smad 2, p-Smad 2 and follistatin antibodies, confirming the presence of myostatin, its downstream Smad signaling pathway proteins and its natural inhibitor in both control and ESLUTD tissue samples. The expression levels of myostatin, Smad2 and p-Smad 2 proteins were higher in ESLUTD muscle samples as compared to the control (Figure 2C).

### 2.3. Expression of Essential Contractile Markers in Bladder-Derived Smooth Muscle Cells

**Figure 3:**
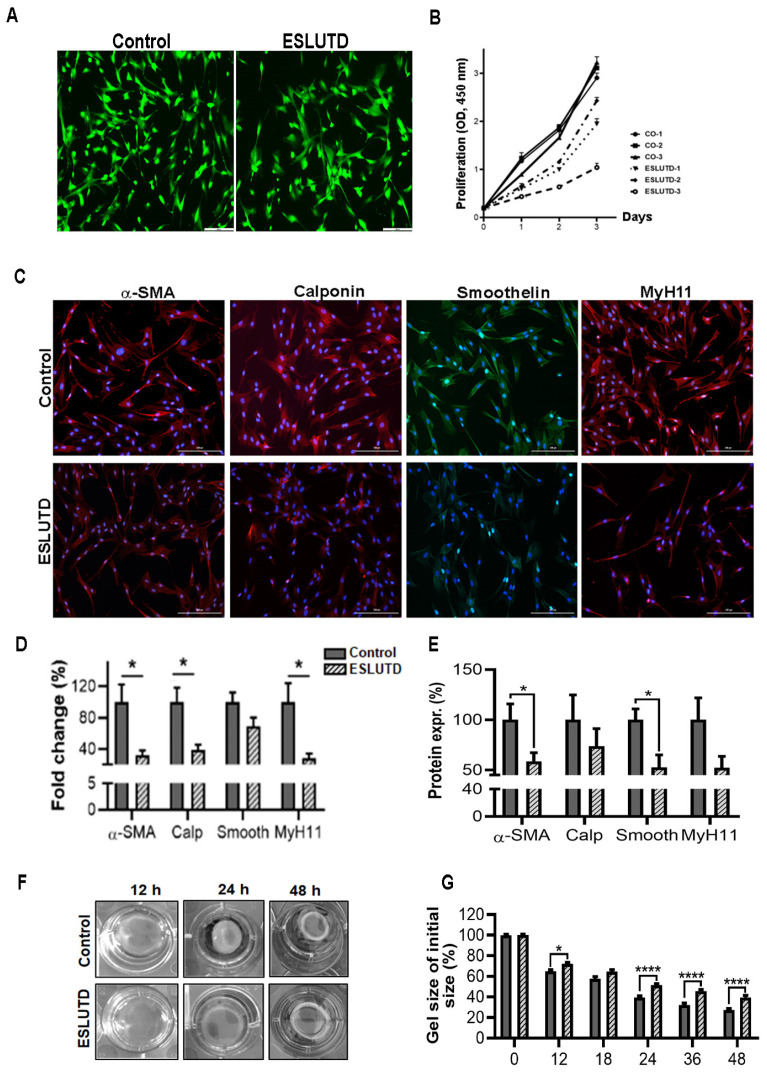
Assessment of contractile phenotype in hSMCs isolated from control and ESLUTD bladders. (**A**) Microscopic images of hSMCs stained with Calcein AM. Green staining indicates viable cells. Both groups showed an equivalent number of viable cells. Scale bar, 200 µm. (**B**) Cell proliferation rate of control and ESLUTD hSMCs. Control hSMCs had a higher proliferation rate compared to ESLUTD cells measured after 1, 2 and 3 days. Symbols represent the mean ± SD, n = 3 of each group in triplicates. (**C**) Immunofluorescence staining of essential contractile proteins α-SMA (red), calponin (red), smoothelin (green) and MyH11 (red) in control and ESLUTD hSMCs. Cell nuclei in blue; scale bar, 200 μm. (**D**) Quantitative real-time PCR. Fold change in gene expression of α-SMA, calponin, smoothelin and MyH11. (**E**) Quantitative protein expression of α-SMA (46 kDa), calponin (48 kDa), smoothelin (120 kDa) and MyH11 (180 kDa) analyzed by Simple Western (WES). Both PCR and WES showed lower expression of contractile genes and proteins in ESLUTD hSMCs. Bars represent mean ± SEM of values normalized to control hSMCs taken as 100%. Control n = 5; ESLUTD n = 4. Statistical analysis by unpaired *t*-tests; * *p* ≤ 0.05. (**F**) Spontaneous contraction of hSMCs embedded in collagen gels. Images showing spontaneous contraction of hSMCs at different time points (hours) after seeding. (**G**) Quantification of spontaneous contractility was assessed by measuring collagen disc areas. ESLUTD hSMCs exhibited a decreased cell contraction over time compared to controls. Figure shows percentage of decrease in gel size after different time points. * *p* ≤ 0.05, **** *p* < 0.0001; control n = 6; ESLUTD n = 4.

After isolation, hSMCs were examined microscopically using Calcein-AM to ensure that the isolated pediatric hSMCs (passages 1–5) were viable. No differences in cell viability were observed between the control and ESLUTD group (Figure 3A). This finding was consistent with the cell count results using trypan blue, which showed 95–98% viable hSMCs in both control and ESLUTD samples. During the cultivation of hSMCs, (P0-P2) we observed a slower proliferation in ESLUTD-derived cells compared to controls. This was confirmed by cell proliferation by WST-1 cell proliferation assay. Control SMCs showed a higher proliferation rate compared to ESLUTD cells at all experimental time points (Figure 3B). Next, using immunofluorescence staining, we investigated the essential contractile protein expression in control and ESLUTD hSMCs. Both groups showed elongated and spindle-shaped morphology and cytosolic expression of all contractile proteins, confirming the characteristic smooth muscle phenotype and contractile profile (Figure 3C). To investigate the expression of contractile genes, we conducted real-time PCRs. In ESLUTD hSMCs, downregulation of α-SMA (32.09 ± 6.1 % SEM, *p* = 0.018) and the essential contractile genes calponin (38.75 ± 6.7, *p* = 0.010), smoothelin (69 ± 11.2) and MyH11 (28.41 ± 16.5, *p* = 0.018) was observed compared to normal bladder controls (100%) (Figure 3D). Next, we quantified the expression of contractile proteins by automated Western blotting. Consistent with the staining and real-time PCR results, both groups showed the presence of all contractile proteins. All SMC-specific markers were clearly reduced in ESLUTD compared to the control. A significant reduction was observed in the expression levels of α-SMA (58 ± 8.4 SEM, *p* = 0.02) and smoothelin (52.6 ± 12.4, *p* = 0.02), while calponin (73.74 ± 17.4) and MyH11 (52.4 ± 11.2) showed an accentuated decrease in ESLUTD samples compared to the control (Figure 3E). 

Furthermore, we examined the spontaneous contractility of hSMCs in collagen by a gel contraction assay at different time points (0–48 h after seeding) (Figure 3F,G). A reduced gel size of the bioengineered tissue discs was observed in a time-dependent manner in both control and ESLUTD discs. The disc areas decreased over time in both groups, signifying that the cells increased their contractility. However, the contractions of tissue discs containing ESLUTD hSMCs were significantly lower at all measured time points after seeding: 12 h (6.88% ± 2.36, *p* = 0.03), 24 h (11.95% ± 2.10 *p* < 0.0001), 36 h (13.74% ± 2.48, *p* < 0.0001) and 48 h (12.17% ± 2.48, *p* < 0.0001) compared to control (Figure 3F,G).

### 2.4. Myostatin Expression and Involvement of Smad Pathway in Bladder Smooth Muscle Cells

**Figure 4:**
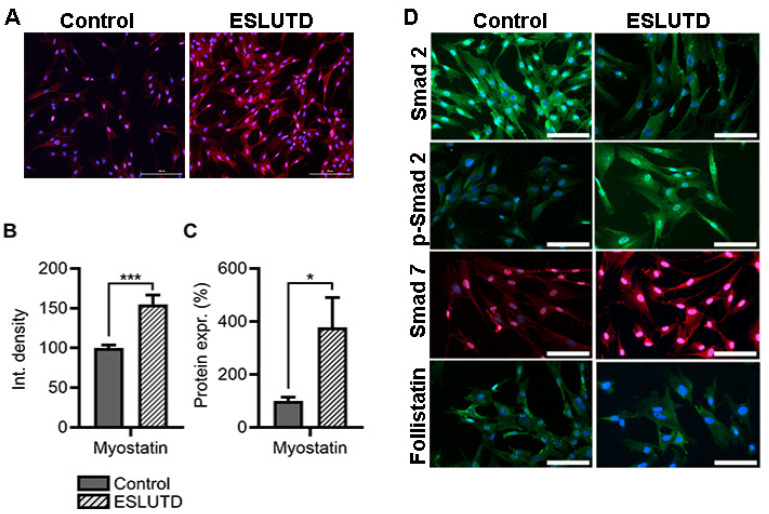
Myostatin signaling pathway protein expression in hSMCs. (**A**) Representative fluorescence images of control and ESLUTD hSMC with myostatin antibody (red), scale bar 100 μm. (**B**) Quantification of microscopic images showed a significant increase in myostatin expression in ESLUTD compared to control. Positive myostatin immunostaining in ESLUTD was measured and compared to control (100%). Bars represent mean ± SEM, n = 4 for each group and 8 images/images. Statistical analysis with unpaired *t*-test; *** *p* ≤ 0.001. (**C**) Quantification at protein level by WES showed significantly higher expression of myostatin compared to control hSMCs (100%) * *p* ≤ 0.05. (**D**) Myostatin downstream proteins Smad 2 (green), p-Smad 2 (green), Smad 7 (red) and natural myostatin inhibitor follistatin (green). Nuclei were stained with DAPI (blue). Scale bar 200 μm.

**Figure 5:**
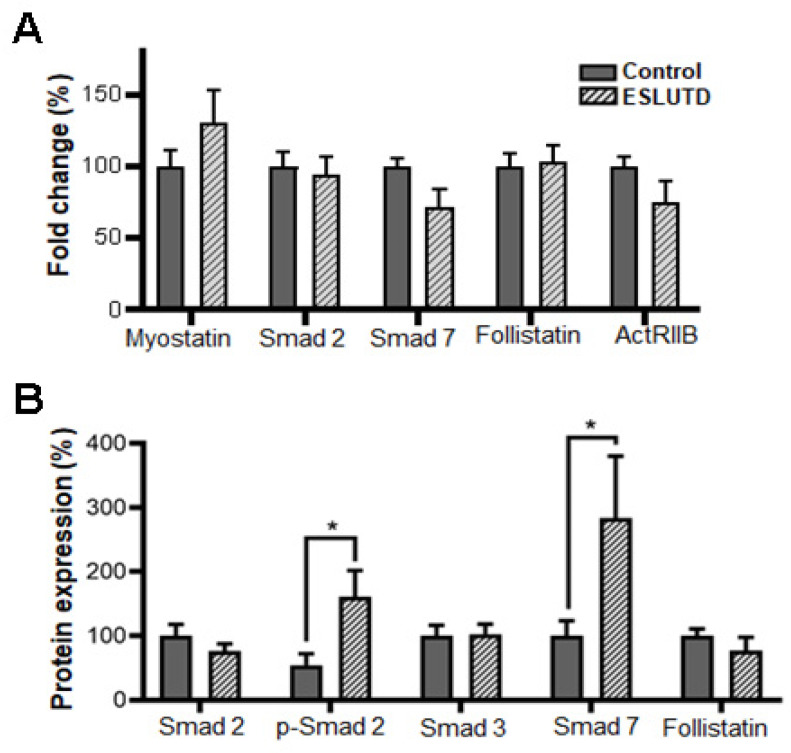
Myostatin signaling pathway expression in control and ESLUTD hSMCs. (**A**) The relative gene expression of myostatin and its signaling pathway Smad 2, Smad 7, follistatin and ACTRIIB in control and ESLUTD hSMCs analyzed by real-time PCR. Bars represent mean ± SEM, n = 4, repeated two times in triplicate. Data analysis by unpaired *t*-tests. (**B**) Protein analysis of myostatin (48 kDa), its pathway proteins Smad 2 (62 kDa), p-Smad 2 (62 kDa), Smad 3 (40 kDa), Smad 7 (48 kDa) and follistatin (48 kDa) analyzed by Simple Western. Bars represent mean ± SEM, n = 5 control and n = 4 ESLUTD samples (in duplicates) were normalized to control taken as 100%. Data analysis by unpaired *t*-tests. * *p* ≤ 0.05.

To investigate the presence and role of myostatin, we examined its expression in hSMCs derived from the healthy bladder and ESLUTD bladder tissues. Myostatin was found in the cytoplasm of hSMCs derived from both control and ESLUTD detrusor muscle by immunofluorescence (Figure 4A). To confirm the visual observation, we quantified the fluorescent intensity in both groups. We detected significantly higher myostatin levels in ESLUTD cells (55.07% ± 9.50, *p* = 0.0002) compared to the control (Figure 4A,B). These results were further confirmed by immunoblotting, where higher expression levels of the myostatin protein were detected in ESLUTD (Figure 4C). We subsequently investigated the involvement of the Smad signaling pathway in hSMCs. By immunofluorescence, we were able to demonstrate the presence of the Smad pathway proteins Smad 2, p-Smad 2, Smad 7 and the natural inhibitor follistatin in both hSMCs groups (Figure 4D). Lower expression levels of Smad 2 and upregulation of p-Smad2 were observed in ESLUTD samples compared to the control (Figure 4D). Furthermore, on the gene level, an increased myostatin expression was detected in ESLUTD cells, while Smad 7 and ActRIIB genes tended to be increased in control hSMCs. No changes in Smad 2 and follistatin gene expression were observed between the groups (Figure 5A). These data were further confirmed by WES analysis, showing a significantly increased expression of p-Smad 2, Smad 7 and a decrease in Smad 2 and follistatin proteins in ESLUTD hSMCs (Figure 5B). 

## 3. Discussion

To our knowledge, this is the first evidence of myostatin in bladder smooth muscle tissue and cells derived thereof. We have shown that myostatin is not only expressed in bladder tissue and isolated hSMCs from healthy children and children with ESLUTD, but also that there are significant differences between the groups. We showed upregulation of myostatin at both gene and protein levels in ESLUTD tissue and cells compared to control bladder samples. These observations correspond well with the reported profibrotic role of myostatin in skeletal and cardiac muscles [32,33]. Our study highlighted an important finding to better understand the pathophysiology behind the structural and functional changes in ESLUTD. 

Our detection of structural changes and the increase in collagen-to-smooth muscle ratio in ESLUTD samples is in line with several previous findings, in which fibrosis in pediatric ESLUTD bladder tissue samples was reported and compared to control samples [34,35,36]. In addition, our data suggests a decrease in cell proliferation and a significant downregulation of the main contractile genes calponin and MyH11, while smoothelin expression was only slightly reduced in ESLUTD tissue compared to healthy controls. The contractile proteins, which are specifically for the functionality of smooth muscle tissues and cells, were expressed in both control and ESLUTD hSMCs. However, all contractile proteins were downregulated in ESLUTD cells compared to the control. A significant decrease was detected in the general smooth muscle marker α-SMA as well as the late contractile marker smoothelin. These findings are consistent with our previous study, in which decreased calponin as well as smoothelin gene and protein expression in hSMCs from children with ESLUTD was observed [37]. The changes in SMC phenotype are linked to a continuous transition between a normal contractile and a synthetic proliferative phenotype [8]. Our results are consistent with the results obtained in an atherosclerosis study, where a correlation of the synthetic phenotype to a decrease in contractile markers in SMCs was shown [8,9]. However, Beamish et al. claimed that the synthetic SMC phenotype is paired with a higher proliferation rate [9], which we did not detect in ESLUTD hSMCs. On the contrary, we observed a decreased proliferation rate in ESLUTD compared to control cells. Although the primary cultures are prone to the phenotypic switch and ESLUTD cells showed a lower contractile phenotype, we believe that these cells remain useful for autologous cell therapies and TE purposes because the contractile proteins α-SMA, calponin, smoothelin and MyH11 are expressed. 

In ESLUTD cells we detected a lower proliferation rate but an increased expression of myostatin at both gene and protein levels. This correlates well with the studies showing an upregulation of myostatin in several diseases such as HIV-infected men with muscle wasting [38], heart failure [39], benign tumors of smooth muscle (leiomyoma) [13] and vascular SMCs [40]. Furthermore, myostatin has been found to be expressed in the cardiac muscle, where it exerts a vital function by sustaining cardiac energy homeostasis and preventing heart failure [41], and where it is upregulated in cardiomyocytes upon myocardial infarction [11]. Moreover, recent studies using clinical samples showed that myostatin may play an important role in the pathogenesis of several reproductive disorders such as uterine myoma, ovary hyperstimulation syndrome, and polycystic ovarian syndrome [42]. Hypothetically, myostatin might not only be increased in ESLUTD but also be a marker for the severity of the dysfunction. However, further research has to be conducted to evaluate this hypothesis. 

**Figure 6:**
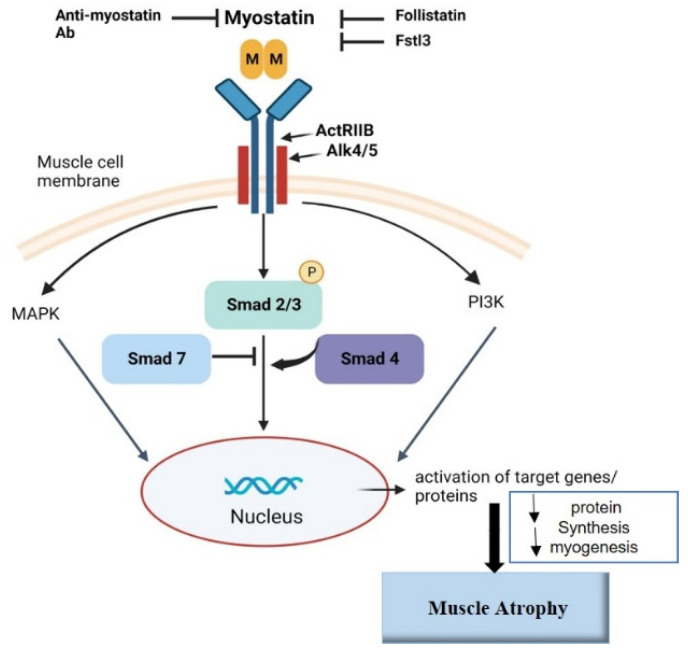
Myostatin signaling pathway. Myostatin is secreted as a propeptide and cleaved by proteases to mature myostatin. The active myostatin dimer binds to ActRIIB leading to phosphorylation and dimerization of ALK4 and/or ALK5. Among others, the Smad pathway is activated which involves the phosphorylation of Smad 2/3 forming a complex with Smad 4. This complex translocates to the nucleus and activates or inhibits target genes [10,15].

In skeletal muscle, myostatin is known to stimulate fibrosis [43] and, therefore, might be partly responsible for increased collagen deposits in detrusor smooth muscle tissue in bladder disorders. At the gene level, expression of the myostatin receptor ActRIIB was downregulated in ESLUTD compared to control samples. Our results suggest a correlation between the increase in myostatin and the reduction in its receptor. After myostatin binds to its receptor, Smad 2 is phosphorylated to p-Smad 2. Therefore, our hypothesis states that an increase in myostatin leads to less Smad 2 and more p-Smad 2 in ESLUTD cells. At the protein level, an increase in p-Smad 2 led to a reduction in the Smad 2 protein, which indicates increased phosphorylation in ESLUTD cells. We did not find any differences in Smad 3 protein expression in both groups. A possible explanation might be that myostatin does not influence the Smad 3 protein to the same extent as Smad 2 in hSMCs. In addition, we found an increase in Smad 7 protein in ESLUTD SMCs. Smad 7 inhibits phosphorylation of Smad 2 and Smad 3, its expression is induced by high concentrations of myostatin via a negative feedback loop [13]. This negative feedback loop could potentially be activated in ESLUTD hSMCs by increased levels of myostatin [16]. The follistatin gene expression remained the same in both the groups, while protein analysis showed a trend towards a decrease in follistatin in ESLUTD hSMCs. It is known that follistatin has an inhibitory effect on myostatin [31,44], but the underlying correlation is still unknown. Patients with Duchene muscular dystrophy had lower levels of follistatin and higher levels of myostatin when treated with steroids, while the opposite was found in steroid-native patients [45]. This suggests an inverse correlation between follistatin and myostatin, which is in line with our findings. Another study on leiomyomas showed increased myostatin levels in cancer, but follistatin expression was unaffected compared to healthy controls [13]. To date there are no consistent results regarding increased or decreased follistatin expression in diseases, thus, further investigations regarding its role are required (Figure 6). Myostatin inhibition can be achieved by blocking the receptor or by blocking myostatin itself. Novartis developed an activin receptor antibody called BYM338 that prevents myostatin- or activin A-induced muscle atrophy through inhibition of Smad2/3 phosphorylation [46]. BYM338 enhanced the differentiation of primary human skeletal myoblasts and increased skeletal muscle mass in mice [46]. Eli Lilly & Company [47] as well as Pfizer [48] developed myostatin antibodies that neutralize the activity of the myostatin protein and, therefore, increase muscle growth and differentiation. These antibodies are still in clinical trials up to phase III and some are therefore not available. 

Using a gel contraction assay, we showed a significant decrease in spontaneous contractility of ESLUTD compared to control hSMCs. This might be due to the decreased expression of smooth muscle-specific contractile markers observed in these diseased hSMCs. Therefore, this strengthens our previous findings showing that ESLUTD hSMCs express a less functional smooth muscle contraction phenotype. This is consistent with the results of Lin et al., who showed that neuropathic hSMCs are not able to contract as well as the corresponding control cells in vitro [49]. 

Consequently, a treatment to improve the quality and quantity of bladder-derived smooth muscle cells is urgently needed. Since myostatin signaling pathways are involved in a wide range of developmental and pathological processes, the targeting of these pathways as a therapeutic strategy to overcome bladder deterioration may be a viable treatment option. As a main focus of our laboratory, our study was carried out using pediatric bladder tissues and cells and not adult cells. Children present a specific challenge, as their diseases and capabilities progress with age. Whether the result of the pediatric group can be translated into adult cells remains to be determined. Whether the myostatin regulatory pathway operates the same way in cells derived from adults suffering from neurogenic detrusor overactivity after spinal cord injury is a topic of future research. 

In the near future, it may be possible to treat smooth muscle-related diseases by inhibiting the expression and function of myostatin in the bladder, but it is essential to note that the inhibition of myostatin may lead to increased muscle mass. However, as research continues, we will learn more about myostatin and its role in bladder-derived smooth muscle in health and disease.

## 4. Materials and Methods

### 4.1. Patient Selection and Biopsies

Bladder tissue biopsies were obtained from pediatric patients during scheduled urological procedures at the University Children’s Hospital Zurich. The procedure was approved by the institutional ethics committee (BASEC-Nr. 2016-01287) and biopsies were taken after informed consent. Bladder samples were taken from children with healthy bladders as control (mean age; 3.1 ± 2.3 years, n = 12.0) without evidence of bladder dysfunction and from children with ESLUTD (mean age; 10.25 ± 3.0 years, n = 4). The detrusor muscle was separated from the urothelium layer by the surgeon. 

### 4.2. Isolation of Human Bladder Smooth Muscle Cells

The muscle tissue from each individual was divided into two pieces, of which one part was used for SMC isolation. Primary SMCs were isolated by a digestion method [50]. Bladder tissues were rinsed in PBS buffer containing 3% Penicillin/Streptomycin (P/S) and 1.5% Fungizone. The tissue was cut into small pieces and incubated in 0.2% collagenase (Sigma-Aldrich, St. Louis, MO, USA) and 0.4% dispase (Gibco, Grand Island, NY, USA) at 37 °C, 5% CO_2_ for 1 h. Full growth medium DMEM/F-12 supplemented with 10% FBS, 1% P/S, 0.5 ng/mL human basic fibroblast growth factor (hbFGF; Sigma-Aldrich, Buchs, Switzerland), 5 ng/mL human epidermal growth factor (hEGF; Sigma-Aldrich, Switzerland) and 5 μg/mL human insulin (Sigma-Aldrich, Switzerland) was added to stop the enzymatic reaction. After removing undigested tissue by filtering through a 100 μm nylon strainer, the samples were centrifuged at 1500 rpm for 5 min and the cells were seeded in full growth medium. 

### 4.3. Tissue Sample Preparation and Histological Analysis

The other half of the smooth muscle tissue was fixed in 4% paraformaldehyde (Artechemis, Zofingen, Switzerland) until paraffin embedding. The paraffin sections were prepared (5 μm) and further processed. Hematoxylin and Eosin (H&E, vector laboratories, CA, USA) and Masson’s Trichrome (Abcam, ab150686, Cambridge, UK) staining were conducted according to the manufacturer’s protocols. Histological analysis was performed using the ImageJ processing software version 8. 

### 4.4. Immunofluorescent Staining 

Paraffin-embedded detrusor muscle samples were de-paraffinized with xylene and rehydrated via a graded series of ethanol. The indirect immunostainings of the tissue sections were performed at 4 °C overnight using the primary antibodies for the contractile proteins anti-α-SMA (1:200, NBP2-33006, Novus biologicals, CO, USA), anti-calponin (1:200, C2687, Sigma Aldrich, Buchs, Switzerland), anti-smoothelin (1:200, NBP2-37931, Novus biologicals, Centennial, CO, USA), anti-MyH11 (1:5, sc-6956, Santa Cruz, Heidelberg, Germany) as well as for the myostatin pathway-related proteins anti-myostatin (1:100, ab201954, Abcam, Cambridge, UK), anti-Smad 2 (1:100, NBP2-33006, Novus biologicals, Centennial, CO, USA), anti-p-Smad 2 (NBP2-54771, Novus biologicals, Centennial, CO, USA) and anti-follistatin (1:100, NBP1-57997, Novus biologicals, Centennial, CO, USA). The slides were incubated with the secondary antibodies sheep anti-mouse Cy3 (1:700, C2181, Sigma Aldrich, Switzerland) and goat anti-rabbit FITC (1:500, FP-SA500 Alexa 488, Brunschwig, Basel, Switzerland) and counter-stained with DAPI (4′,6-diamidino-2-phenylindole, 1:200, D9542, Sigma Aldrich, Buchs, Switzerland). For negative controls, the primary antibody was omitted. For the staining of cells, SMCs were grown and fixed on 4-well glass chamber slides with 4% paraformaldehyde. The indirect immunostainings of the tissue sections and slides were performed at 4 °C overnight using the same primary and secondary antibodies as listed above. For negative controls, the primary antibody was omitted. Images were taken with a Leica Thunder fluorescence microscope (DMi8 Leica Microsystems, Wetzlar, Germany).

### 4.5. Cell Proliferation

To measure SMC proliferation, the cells were seeded on 96-well plates. At indicated time points of 1,2 and 3 days, SMCs were incubated with WST-1 proliferation reagent (Roche Applied Science, Indianapolis, IN, USA) for 3 h at 37 °C, with 5% CO_2_. Cell proliferation was indirectly measured via the color change of the supernatant at 450 nm (EPOCH2 microplate reader, BioTek, Winooski, VT, USA).

### 4.6. Quantitative Real-Time PCR

RNA was isolated using the ReliaPrep RNA Cell Miniprep System (TermoFisher, Waltham, MA, USA) according to the manufacturer’s protocol. The total RNA was measured and reverse transcribed with random primers (High-Capacity cDNA reverse transcription, Applied Biosystems, Bedford, MA, USA). For PCR analysis, the ThermoFisher primers for contractile genes α-SMA (Hs05005341_m1), calponin (Hs00154543_m1), smoothelin (Hs00199489_m1), MyH11 (Hs00224610_m1) and primers for myostatin pathway-related genes myostatin (Hs00976237_m1), Smad 2 (Hs00183425_m1), Smad 7 (Hs00998193_m1), follistatin (Hs00246256_m1) and ActRIIB (Hs00609603_m1) were used. The primer for GAPDH (Glycerinaldehyde-3-phosphate-dehydrogenase, 4333764T) was used as an internal control. Cycle threshold (CT) values were measured and fold changes were calculated either with the 2^−ΔΔCt^ or 2^−ΔCt^ method.

### 4.7. Fluorescence-Activated Cell Sorting

For flow cytometry analysis, SMCs were labeled with primary antibodies for anti-α-SMA (1:200, NBP2-33006, Novus biologicals, Centennial, CO, USA), anti-calponin (1:150, C2687, Sigma Aldrich, Buchs, Switzerland), anti-myostatin (1:50, H00002660-M07, Novus biologicals, Centennial, CO, USA) or anti-Isotype IgG mouse (1:150, sc-2877, Santa Cruz, Heidelberg, Germany). The cells were incubated with the secondary antibodies anti-mouse FITC (1:200, BD 55988, BD Biosciences, Allschwil, Switzerland) or anti-rabbit FITC (1:200, FP-SA5000 Alexa 488, Brunschwig, Switzerland). Analysis was conducted using an LSR Fortessa^TM^ cell analyzer (BD Bioscience, Allschwil, Switzerland) and results were analyzed with FlowJo software 7.2.4 (Tree Star Inc., Ashland, OR, USA). 

### 4.8. Immunoblotting (Automated Western Blotting—WES)

The total protein concentration was measured using a BCA protein assay kit (Thermo scientific, Lausanne, Switzerland). A total of 1–1.5 mg/mL protein was used for WES analysis using a 12–230 kDa cartridge kit (Protein Simple WES, Germany). The primary antibodies for the contractile proteins anti-α-SMA (1:100, NBP2-33006, Novus biologicals, Centennial, CO, USA), anti-calponin (1:100, C2687, Sigma Aldrich, Buchs, Switzerland), anti-smoothelin (1:50, NBP2-37971, Novus, Centennial, CO, USA), anti-MyH11 (1:50, NBP2-44533, Novus biologicals, Centennial, CO, USA) as well as for the myostatin pathway-related proteins anti-myostatin (1:50, ab201954, Abcam, Cambridge, UK), anti-Smad 2 (NBP2-67376, Novus biologicals, Centennial, CO, USA), anti-p-Smad 2 (1:50, NBP2-54771, Novus biologicals, Centennial, CO, USA), anti-Smad 3 (1:50, NB100-56479, Novus biologicals, Centennial, CO, USA), anti-Smad 7 (1:50, MAB2029, Novus biologicals, Centennial, CO, USA) and anti-follistatin (1:50, AF669, R&D Systems, Minneapolis, USA) were used. Anti-GAPDH (1:100, B300-221, Novus Biologicals, Centennial, CO, USA) served as an internal control. Samples were analyzed using Compass software (ProteinSimple, version 6.1.0, Wiesbaden, Germany). Electropherogram and virtual blot were checked and evaluated for each sample. A chemiluminescent signal was quantified by the software and the area of each sample was normalized to GAPDH.

### 4.9. Gel Contraction Assay

To measure the spontaneous contraction of the detrusor SMCs, small in vitro tissue-like collagen discs were formed by mixing SMCs (0.5 × 10^6^ cells/mL) with 3 mg/mL rat tail collagen type 1 (BD Biosciences, 354236, Allschwil, Switzerland) and contraction assays were performed [51]. The floating collagen discs were incubated at 37 °C, 5% CO_2_ for 12, 24 and 48 h. Auto contraction of the collagen discs was excluded by cell-free analysis. The contraction was documented by pictures taken from a fixed distance and the areas of the disks were measured with ImageJ software. 

### 4.10. Statistical Analysis

Results were presented as mean with corresponding standard error of the mean (±SEM) and analyzed by an unpaired *t*-test or one-way ANOVA with Dunnett’s post-correction using GraphPad Prism version 8.0.0. (GraphPad Software, Inc., La Jolla, CA, USA). *p*-values < 0.05 were considered statistically significant. 

## 5. Conclusions

Our study shows for the first time the presence of myostatin in bladder tissue and derived smooth muscle cells. Upregulation of myostatin and changes in the Smad pathway were observed in ESLUTD patient samples. This represents an important finding to better understand the pathophysiology behind the structural and functional changes in ESLUTD. In addition, myostatin inhibitors may have therapeutic potential in ESLUTD and other smooth muscle disorders. However, further research has to be conducted to confirm this hypothesis.

## Data Availability

The datasets generated and analyzed during the current study are not publicly available. However, they are available from the corresponding author upon reasonable request.
